# Prognostic Gene Expression, Stemness and Immune Microenvironment in Pediatric Tumors

**DOI:** 10.3390/cancers13040854

**Published:** 2021-02-18

**Authors:** David Stahl, Rainer Knoll, Andrew J. Gentles, Christian Vokuhl, Andreas Buness, Ines Gütgemann

**Affiliations:** 1 Institute of Pathology, University Hospital Bonn, 53127 Bonn, Germany; david.stahl1@uk-koeln.de (D.S.); rainer.knoll@uni-bonn.de (R.K.); christian.vokuhl@ukbonn.de (C.V.); 2 Department I of Internal Medicine, University Hospital of Cologne, 50937 Cologne, Germany; 3 Department of Medicine (Biomedical Informatics), Stanford University, Stanford, CA 94035, USA; andrewg@stanford.edu; 4 Institute for Medical Biometry, Informatics and Epidemiology, Medical Faculty, University of Bonn, 53127 Bonn, Germany; andreas.buness@uni-bonn.de; 5 Institute for Genomic Statistics and Bioinformatics, Medical Faculty, University of Bonn, 53127 Bonn, Germany

**Keywords:** CIBERSORT, tumor-infiltrating immune cells, tumor immune microenvironment, pediatric tumors, stemness, prognostic gene expression

## Abstract

**Simple Summary:**

Tumors in children and young adults are rare and diagnostically distinct from those occurring in older patients. They frequently arise from developing cells, resembling stem cells, which may explain some of the clinical and biologic differences observed. The aim of this retrospective transcriptome study was to investigate the prognostic landscape, immune tumor microenvironment (TME) and stemness in a cohort of 4068 transcriptomes of such tumors. We find that patients’ prognosis correlates with distinct gene expression patterns similar to adult tumor types. Stemness defined by a computational stemness score (mRNAsi) correlates with clinical and molecular parameters that is distinct for each tumor type. In Wilms tumors that recapitulate normal kidney development microscopically, stemness correlates with distinct patterns of immune cell infiltration by transcriptome analysis and by cell localization in tumor tissue.

**Abstract:**

Pediatric tumors frequently arise from embryonal cells, often displaying a stem cell-like (“small round blue”) morphology in tissue sections. Because recently “stemness” has been associated with a poor immune response in tumors, we investigated the association of prognostic gene expression, stemness and the immune microenvironment systematically using transcriptomes of 4068 tumors occurring mostly at the pediatric and young adult age. While the prognostic landscape of gene expression (PRECOG) and infiltrating immune cell types (CIBERSORT) is similar to that of tumor entities occurring mainly in adults, the patterns are distinct for each diagnostic entity. A high stemness score (mRNAsi) correlates with clinical and morphologic subtype in Wilms tumors, neuroblastomas, synovial sarcomas, atypical teratoid rhabdoid tumors and germ cell tumors. In neuroblastomas, a high mRNAsi is associated with shortened overall survival. In Wilms tumors a high mRNAsi correlates with blastemal morphology, whereas tumors with predominant epithelial or stromal differentiation have a low mRNAsi and a high percentage of M2 type macrophages. This could be validated in Wilms tumor tissue (*n* = 78). Here, blastemal areas are low in M2 macrophage infiltrates, while nearby stromal differentiated areas contain abundant M2 macrophages, suggesting local microanatomic regulation of the immune response.

## 1. Introduction

Stemness, defined as the potential to self-renew and differentiate from a cell of origin, is a feature of precursor cells in the developing embryo [1]. Pediatric tumors are distinct from adult tumors in that they frequently arise from prenatal embryonal or early postnatal cells [2]. Recent studies [3,4] have provided means to infer tumor stemness from transcriptome data using computation of a stemness index score (mRNAsi). Several stemness indices have been used in the past [3,4,5,6] and cross-validated in adult tumor types, showing similar results [3]. However, what has been lacking is a direct comparison of stemness and immune cell infiltration in tumors of the pediatric and mostly young adult age.

One of the explanations as to why pediatric tumors are less accessible for elimination by the immune system is stemness itself. The mRNAsi has been linked with immune cell exclusion and protection of antigenic clones from elimination [3,4,7]. Embryonic, mesenchymal and induced pluripotent stem cells possess immune modulatory properties, while resistance to immune-mediated destruction is an intrinsic feature of quiescent adult tissue stem cells [8] and cancer stem cells [9,10,11]. As these findings imply that immune control may be improved in tumors by targeting the stemness phenotype, we were interested to examine the relation of stemness and immune tumor microenvironment (immune TME) in pediatric tumors in a more systematic fashion.

Tumors in children and young adults are histologically diverse; tumors of the Ewing´s sarcoma family of tumors (ESFT) and neuroblastomas (NB) are mainly composed of immature cells resembling stem cells (“small round blue cell tumors“) [12,13], while others, especially those that have a better prognosis, show various degrees of differentiation, such as Wilms tumors (WILMS). In WILMS, the resemblance to embryonal tissue in some cases is striking, with blastemal immature stem cell-like tumor areas and more differentiated areas, “epithelial” mimicking normal renal tubules and “stromal” mimicking mature stromal elements all in one tumor [14,15]. Thus, this tumor type is well suited to study the correlation of stemness and immune TME on a microscopic level.

Immunotherapies are currently evaluated in pediatric tumor patients; however, these avenues are facing problems due to a lack of PD-L1 expression in some tumors [16,17], immune suppressive macrophage infiltration [18] or, as recently shown in sarcomas, with only a small fraction of tumors containing tertiary lymphoid structures necessary for a treatment response [19]. Neoantigens that can be recognized by the immune system may be too sparse, because pediatric tumors are genetically less complex than adult cancers [20,21] and, in about 8%, occur with concurrent germ line mutations [22,23].

In multiple solid tumors, “cancer stem cells” defined as self-renewing cells facilitating tumor initiation and promoting metastasis and therapeutic resistance have been identified that reciprocally interact with immune cells, including tumor associated macrophages [24].

Previous in silico transcriptome studies in cancers occurring predominantly in older individuals identified oncogenic dedifferentiation to be associated with stemness and correlated stemness with immune checkpoints and infiltrating immune cells [4]. Another pan-cancer analysis in 21 solid tumor entities discovered negative associations between antitumor immunity and stemness, and positive associations between stemness and intratumoral heterogeneity [3]. The same authors presented evidence that stemness is strongly associated with cell-intrinsic suppression of endogenous retroviral expression and type I interferon signaling [3]. Furthermore, increased stemness was associated with strong polarization of infiltrating leukocyte populations toward a macrophage-dominated, CD8+ T cell-depleted composition for most cancers.

Previously, pediatric tumors of the central nervous system have been most thoroughly investigated regarding the immune TME [25]. In particular, medulloblastomas (MB), malignant rhabdoid tumors and pediatric high grade gliomas (HGG) showed significant associations regarding immune profiles with molecular subgroups and prognosis. Recently comparison of stemness and the immune TME in MB showed an inverse correlation of stemness with immunosuppressive M2 type macrophages [26].

In this study, we assembled, curated and integrated, in total, 4068 cancer gene expression profiles (GEPs) and clinical data including outcome data (overall survival, OS) from the public domain Gene Expression Omnibus (GEO) confined to 12 tumor entities occurring mostly in children and young adults with sufficient and comparable data sets: acute lymphatic leukemia (ALL), atypical teratoid rhabdoid tumor (ATRT), Ewing’s sarcoma family of tumors (ESFT), germ cell tumors (GCT), high grade gliomas (HGG), medulloblastomas (MB), neuroblastoma (NB), osteosarcoma (OSTEO), CNS-PNETs, rhabdomyosarcoma (RMS), synovial sarcomas (SS) and Wilms tumors (WILMS). 

A subcohort of 3094 transcriptomes with complete OS data was used to assess the prognostic landscape of gene expression in comparison to that of adult tumors using the PRECOG algorithm (“PRECOG-cohort”, Table S1) [27]. 

In order to determine the composition of immune cell infiltration by transcriptome analysis, we next used the CIBERSORT algorithm on a subcohort of 1883 transcriptomes with more complete clinic-pathologic data (“CIBERSORT-cohort”, Table S2). CIBERSORT reconstructs the type and quantity of immune cell subsets in bulk tumor tissue using an expression matrix derived from gene expression data of 22 known immune cells from the peripheral blood using 547 characteristic marker genes [28]. This algorithm allows the identification of even rare and functionally distinct immune cell types (e.g., mast cells, gamma delta T-cells, memory B-cells, regulatory T-cells etc.) in sparse tissue samples.

The same cohort was used to determine stemness features using the mRNAsi index, a novel stemness index for evaluating the dedifferentiation potential of tumor cells [4].

This retrospective in silico analysis shows that prognostic gene expression, stemness and immune cell infiltration in pediatric tumors is similar to that in adult tumor types; however, within the pediatric group of tumors, distinct for each entity. Finally, we validate some of the findings in WILMS by immunohistochemistry.

## 2. Results

### 2.1. The Prognostic Landscape of Gene Expression

**Figure 1:**
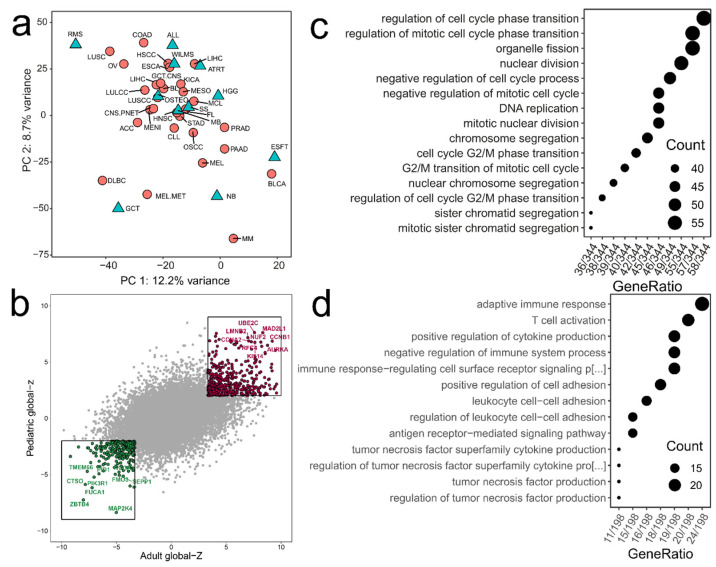
Prediction of clinical outcomes from genomic profiles (PRECOG) in adult versus pediatric tumors (**a**) Principal component analysis (PCA). Comparison of the prognostic landscape (meta-z-scores) of 34 adult tumors (red circles) [27] and 12 tumors occurring in children and young adults (“pediatric tumors“, cyan triangles) (“PRECOG-cohort”, *n* = 3094). Tumors with similar histogenesis, such as neuroblastoma, melanoma and melanoma metastases cluster closely together, indicating a trend for similar gene prognostic associations. (**b**) Dot plot comparing global meta-z-scores (PRECOG) of pediatric (*n* = 12) versus adult malignancies (*n* = 34) [27]. The correlation was significant (Pearson r = 0.39; *p* < 2.2 × 10^−16^). Top favorable prognostic genes in green, top adverse prognostic genes in red. Full list pediatric global meta-z-score and individual meta-z-scores for each gene: Table S3. (**c**,**d**) Gene ontology (GO) enrichment of pediatric adverse (c) and favorable (**d**) prognostic genes. The dot plot of pediatric vs. adult global z-values (**b**) was subdivided into sectors, upper right sector (red): genes adverse prognostic in pediatric and adult tumors, lower left sector (green): favorable prognostic genes in pediatric and adult tumors. GO enrichment was performed on the genes of the respective sector using the GO biological processes database. Dot plots show significantly enriched terms, defined by a Bonferroni corrected *p*-value < 0.05 and a FDR < 0.05. Abbreviations: Pediatric tumors: atypical teratoid rhabdoid tumor, ATRT; Ewing’s sarcoma family of tumors, ESFT; germ cell tumors, GCT; high grade gliomas, HGG; medulloblastomas, MB; neuroblastoma, NB; osteosarcoma, OSTEO; CNS-PNETs; rhabdomyosarcoma, RMS; synovial sarcomas, SS; Wilms tumors, WILMS. Adult tumors: adrenocortical cancer, ACC; bladder cancer, BLCA; astrocytoma, ASTR; glioblastoma, GBM; glioma, LGG; meningioma, MENI; breast cancer, BRCA; colon cancer, COAD; gastric cancer, STAD; head and neck cancer, HNSC; hypopharyngeal cancer, HSCC; esophageal cancer, ESCA; oral SCC, OSCC; AML, LAML; Burkitt lymphoma, BL; CLL, CLL; DLBCL, DLBC; follicular lymphoma, FL; mantle cell lymphoma, MCL; multiple myeloma, MM; kidney cancer, KICA; liver cancer, LIHC; lung adenocarcinoma, LUAD; large cell lung cancer, LULCC; squamous cell lung cancer, LUSCC; small cell lung cancer, LUSC; melanoma, MEL; melanoma metastasis, MEL MET; mesothelioma, MESO; ovarian cancer, OV; pancreatic cancer, PAAD; prostate cancer, PRAD.

In order to compare “stemness” and immune related gene expression in pediatric tumors, we first compared prognostic gene expression in pediatric (PRECOG-cohort) to that of a previously published cohort of adult tumors [27]. The prognostic landscape in pediatric tumors was quite similar to that in predominantly adult tumors, as demonstrated by principal component analysis (PCA) and dot plot analysis of the “meta z-scores” (Figure 1a,b). Z-scores represent the statistical significance of each gene’s association with survival. To facilitate cross-cancer analyses, z-scores for individual studies were combined to yield meta-z-scores for the prognostic significance of each gene in each cancer type. Shared genes by all cancers (Figure 1b) with an adverse prognosis (high positive global meta-z-scores), such as MAD2L1, CCNB1, NUF2 and UBE2C, were associated with gene ontology (GO) enrichment analysis terms related to proliferation, replication and mitosis, similar to adult tumors [27] (Figure 1c). Likewise, genes with favorable prognosis (negative global meta-z-scores) (e.g., MAP2K4, ZBTB4, FUCA1, SEPP1, TMEM66, CTSO) were associated with adaptive immune response, cell adhesion and T-cell activation (Figure 1d). Tumors with neuroectodermal origin, such as neuroblastoma, melanoma and melanoma metastases cluster closely together based on 21% of the variance of the full data set (Figure 1b). Differences were observed in top adverse and favorable genes expressed per tumor entity (individual meta z-scores for each gene per tumor type and global meta-z-scores for all pediatric tumor types: Table S3; top ten adverse and favorable genes per tumor entity: Table S4).

### 2.2. Immune Cell Infiltration—Composition and Prognostication

**Figure 2:**
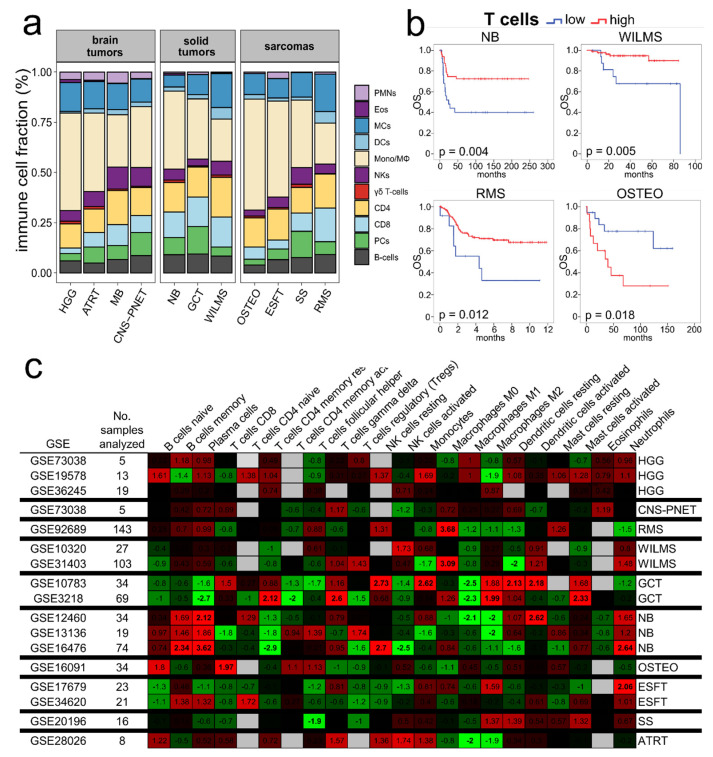
Immune microenvironment in pediatric tumors, determined using CIBERSORT transcriptome analysis. (**a**) Estimated relative fractions of 22 leukocyte subset across 11 pediatric tumors (“CIBERSORT-cohort)”, pooled into 11 immune populations for clarity. (**b**) Prognostic value of tumor infiltrating T-cells. Kaplan–Meier analyses showing survival with low (blue) and high (red) frequency of relative fractions of T-cells (CIBERSORT), as indicated: longer survival was observed in NB (GSE16476, *n* = 74, *p* = 0.004), in WILMS (GSE31403, *n* = 103, *p* = 0.005) and in RMS (GSE92689, *n* = 138, *p* = 0.012), whereas in OSTEO (GSE16091, *n* = 34) higher T-cell content was associated with reduced OS (*p* = 0.018). Cutoff points of cell abundance were set at the point with the most significant separation (log-rank test) as previously described [29]. (**c**) Correlation of CIBERSORT-inferred cell type fractions and overall survival (OS). Heatmap, z-scores from univariate Cox regression on relative fractions of individual immune cell subsets. Green indicating favorable prognostic cell subsets, red indicating adverse prognostic cell subsets. |z| > 1.96 is equivalent to a two-sided *p* < 0.05 and are marked in bold. Abbreviations: atypical teratoid rhabdoid tumor, ATRT; Ewing’s sarcoma family of tumors, ESFT; germ cell tumors, GCT; high grade gliomas, HGG; medulloblastomas, MB; neuroblastoma, NB; osteosarcoma, OSTEO; CNS-PNETs; rhabdomyosarcoma, RMS; synovial sarcomas, SS; Wilms tumors, WILMS.

As the GO enrichment of favorable prognostic genes in pediatric tumors revealed immune response and T-cell activation as significant terms, we applied CIBERSORT [28] next, in order to systematically define the composition of tumor infiltrating immune cells in 1883 pediatric tumors (Table S2). The cell composition of the immune microenvironment varied across different pediatric tumor types, but was similar to adult tumors with the predominant immune cell fraction consisting of macrophages, followed by T-cells (Figure 2a) [27].

Kaplan–Meier analysis revealed that T-cells were associated with a good prognosis in NB, WILMS and RMS (Figure 2b), which was consistent with the results of the GO enrichment analysis and with previous results on the favorable prognostic value of T-cells in ESFT [18]; whereas, surprisingly, T-cells, in particular CD8 T-cells, were associated with a shortened OS in OSTEO (Figure 2b,c). For other cell types, prognostication was not as consistent as previously shown for adult tumors (Figure 2c) [27]. Increased plasma cells were associated with shorter survival in NB, but longer survival in GCT. In NB M2 type macrophages were associated with longer survival, most likely attributable similarity in signature with microglia (Figure 2c). In summary, we found no evidence for a distinct pattern in terms of prognostication or immune cell infiltrates in pediatric tumors, thus next, we looked at stemness and immune cell correlation in more detail.

### 2.3. Stemness Correlates with Immature Histology, Clinic-Pathological Features and Survival

**Figure 3:**
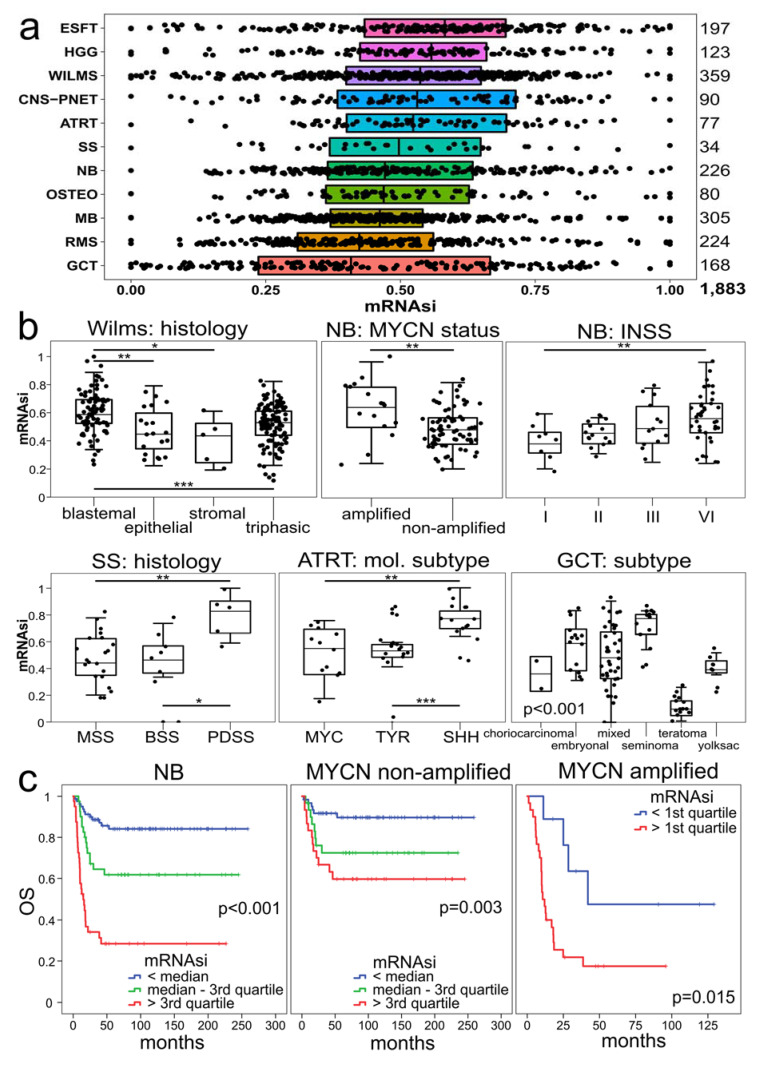
(**a**) Stemness index scores (mRNAsi) across different childhood cancers (CIBERSORT-cohort) ranging from 0 (low) to 1 (high). Dots represent individual pediatric tumors. The median was significantly different between tumor types (*p* < 0.001, Kruskal–Wallis test). (**b**) mRNAsi depends on the histologic, molecular and clinical subtype in WILMS, NB, SS, ATRTs and GCT (individual GSE study sets). Blastemal histology is associated with higher stemness indices compared to other histological subtypes in WILMS (GSE31403, *n* = 220). MYCN-amplified neuroblastomas are associated with higher stemness indices (GSE16476, *n* = 88). Higher mRNAsi indices are found in stage IV NBs compared to lower stages (GSE16476, *n* = 88). Poorly differentiated SS (PDSS) show significantly higher mRNAsi scores than monophasic and biphasic SS (MSS and BSS) (GSE20196, *n* = 34). SHH-ATRTs have higher stemness indices than MYC-ATRTs and TYR-ATRTs (GSE70678, *n* = 42). Seminomas and embryonal GCTs show higher mRNAsi values compared to other GCT subtypes by group variance testing (GSE3218, *n* = 90) (*p* < 0.001, Kruskal–Wallis test). For all other comparisons, a pairwise Mann–Whitney U test was performed, indicated by bars and stars representing the level of significance: * *p* < 0.05, ** *p* < 0.01, *** *p* < 0.001. (**c**) A low mRNAsi is associated with longer OS independent of MYCN amplification. Kaplan–Meier analyses comparing survival with low (blue), intermediate (green) and high (red) mRNAsi values. Left: entire cohort (*n* = 162), middle: MYCN non-amplified (*n* = 122), right: MYCN amplified (*n* = 39) NBs. Abbreviations: atypical teratoid rhabdoid tumor, ATRT; Ewing’s sarcoma family of tumors, ESFT; germ cell tumors, GCT; high grade gliomas, HGG; medulloblastomas, MB; neuroblastoma, NB; osteosarcoma, OSTEO; CNS-PNETs; rhabdomyosarcoma, RMS; synovial sarcomas, SS; Wilms tumors, WILMS; monophasic synovial sarcoma, MSS; biphasic synovial sarcoma, BSS; poorly differentiated synovial sarcoma, PDSS.

We applied the stemness algorithm mRNAsi to the CIBERSORT-cohort. This analysis revealed that the median stemness index varied across pediatric tumor types (*p* < 0.001; Kruskal–Wallis test) (Figure 3a). Within brain tumors, HGG had the highest mRNAsi (median 0.58) followed by CNS-PNET (median 0.53), ATRT (median 0.52) and MB (median 0.46). RMS had a low mRNAsi (median 0.42), consistent with myogenous differentiation in many cases. WILMS showed a high variance of mRNAsi (median 0.54), ranging from high in predominant blastemal (median 0.58) to low mRNAsi in stromal or triphasic differentiated WILMS (median 0.53); thus, this index correlates well with histologic phenotype (GSE1403, *n* = 220) (Figure 3b). This result was confirmed using an independent cohort of WILMS (GSE53224, *n* = 42), where blastemal histology showed higher stemness indices than triphasic histology (*p* = 0.017, Mann–Whitney U test).

Patients with MYCN gain in NB have inferior outcomes, especially in otherwise more favorable groups, including inferior event-free survival and overall survival [30]. We find that MYCN-amplification was associated with higher stemness indices (GSE16476, *n* = 88, *p* = 0.006, Mann–Whitney U test) (Figure 3b). This result was confirmed using three independent cohorts of NBs (GSE12460, *n* = 46, *p* = 0.001; GSE13136, *n* = 30, *p* < 0.001; GSE16237, *n* = 50, *p* = 0.027; Mann–Whitney U test). Furthermore, stage IV NBs correlated with higher mRNAsi scores compared to stage I NBs (GSE16476, *n* = 88, *p* = 0.006, Mann–Whitney U test) (Figure 3b). This data was also confirmed using two independent cohorts of NB (GSE12460, *n* = 42; GSE13136, *n* = 27), where stage I NBs showed lower stemness indices than stage IV NBs (*p* = 0.027, *p* = 0.005, respectively, Mann–Whitney U test). Poorly differentiated SS (PDSS) and SHH-ATRTs showed higher stemness indices compared to other subtypes (GSE20196, *n* = 34; GSE70678, *n* = 42) (Figure 3b). Out of all GCTs seminomas showed the highest mRNAsi (median 0.77) consistent with germ cell morphology. Within the non-seminomatous GCT group, embryonal carcinoma (median 0.59) showed highest mRNAsi scores (GSE3218, *n* = 90, *p* < 0.001, Kruskal–Wallis test) (Figure 3b).

In NBs, the mRNAsi correlated with an increased hazard of death by uni- (*p* < 0.001) and multivariate Cox regression analysis (*p* = 0.04, *n* = 162) (Table S5). A shortened survival was observed with NB cases with a high mRNAsi both in MYCN amplified as well as non-amplified cases by Kaplan–Meier analysis (Figure 3c).

In no other pediatric tumor entity did we find a significant correlation of mRNAsi with patient outcome; however, correlations with stemness and clinical or molecular features could be seen (Figure 3b).

### 2.4. Immune TME and Stemness in WILMS

While most tumor entities examined did not show a correlation of mRNAsi with CIBERSORT fractions, WILMS did show correlations with naïve B-cells, CD8+ T-cells, follicular helper T-cells, regulatory T-cells, monocytes and activated mast cells correlating directly and CD4 memory T-cells, gamma delta T-cells, M0, M1 and M2 type macrophages, resting mast cells and eosinophils correlating indirectly with the mRNAsi (Table S6). One of the reasons may be the wide spectrum of stemness because of differences in histology. In WILMS, the tumor cells recapitulate the normal nephron development from metanephric mesenchyme cells to renal tubular epithelial cells. Even within one tumor often various histologic patterns are observed with blastemal areas, myogenous stroma, and epithelial differentiation. Therefore, WILMS are a suitable neoplasm to examine the immune TME in the context of stemness in situ in more detail.

**Figure 4:**
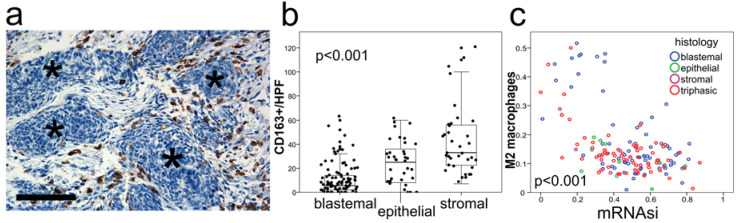
M2 macrophages in WILMS. (**a**) Blastemal tumor areas of WILMS tumors are devoid of M2 macrophages by tumor tissue microarray (TMA) analysis. Representative photomicrograph, CD163 expression by immunohistochemistry in 78 WILMS tumors. Stars (*) indicate blastemal tumor areas (DAB, 400×). Scale bar representing 100 µm. (**b**) Bar graph, low densities of CD163 positive macrophages (immunohistochemistry) in blastemal areas in WILMS tumors compared with epithelial and stromal areas (*p* < 0.001, Kruskal–Wallis test). Macrophages were counted in 2–3 HPFs in 1–2 cores per tumor within blastemal (*n* = 92), epithelial (*n* = 36) and stromal (*n* = 36) tumor areas separately. (**c**) Inverse correlation of the relative frequency of M2 macrophages (CIBERSORT) and mRNAsi in WILMS by transcriptomic analysis. (rho= −0.508, *p* < 0.001, Spearman rank test).

Next, we validated selected results in tumor tissue in situ. Because a high mRNAsi correlated with the blastemal subtype in WILMS by transcriptome analysis (Figure 3b), we used blastemal histology as a microscopic surrogate for stemness. We focused on macrophages enumerated by CIBERSORT, because of their high abundance and easy detection and quantification by immunhistochemistry in situ. Tumor tissue microarrays (TMAs) of 78 WILMS tumors that were annotated as to the histologic pattern per core (1–2 cores per tumor) were analyzed by manual cell counting. We found that blastemal tumor areas were almost devoid of CD163 positive M2 type macrophages, while tumor areas with stromal differentiation showed dense macrophage infiltrates (Figure 4a,b) and areas displaying epithelial differentiation showing an intermediate density (Figure 4b). These observations support the results by retrospective transcriptome analysis, where M2 macrophage frequency determined by CIBERSORT analysis correlated inversely with the computed mRNAsi (Figure 4c).

To conclude, we did not observe a correlation of stemness with distinct patterns in the TME defined by CIBERSORT; however, in individual tumor types stemness correlates with clinic-pathologic parameters and selected immune cell subsets.

## 3. Discussion

Analyzing transcriptomes of 4068 tumors of pediatric and young adult age we can demonstrate that the prognostic landscape and immune microenvironment overall is similar to adult tumors. Stemness (mRNAsi) is associated with immature cell morphology and clinical subtype. Correlations are tumor type specific with NBs demonstrating a positive correlation of stemness with shortened survival and in WILMS stemness correlates with blastemal morphology.

The correlation of a high mRNAsi with an immunosuppressive microenvironment is thought to be a general phenomenon coinciding with dedifferentiation and oncogene expression in tumors [3,4]. In pediatric tumors no distinct pattern of immune cell infiltrates with stemness was observed across all analyzed tumors. Instead, a correlation of stemness with clinic-pathologic parameters and survival within specific diagnostic entities can be seen. These findings are consistent with earlier studies in adult cancers using TCGA data and other stemness indices [3] as well as using the mRNAsi [4].

While various stemness indices have been broadly analyzed in adult tumors [3], this report focuses on tumors occurring predominantly at the pediatric and young adult age. Interestingly, the median of mRNAsi in our cohort is higher than in tumors occurring mostly in older adult patients [4]. This finding is understandable, because some tumors in our list arise prenatally and the mRNAsi is a signature derived from transcriptome profiles of embryonal cells as well as immature precursor cells/stem cells (Figure 3b). It is important to note, however, that the mRNAsi index [4] has been designed including genes regulating proliferation. Other indices have removed such genes showing comparable results [3]. While this approach is difficult, as many genes regulate stemness as well as proliferation, future studies need to address how stemness and proliferation are interconnected in pediatric tumors.

Supporting the mRNAsi as a marker of stemness in pediatric tumors, our data shows a correlation of a high mRNAsi with an immature “precursor cell look” by histology: blastemal tumor types such as predominant blastemal WILMS or neuroblastoma have a higher mRNAsi than tumors showing histologic differentiation, such as teratomas (Figure 3b).

In WILMS, immune cell infiltrates determined by CIBERSORT did correlate with stemness (mRNAsi), whereas other tumor types did not show a direct correlation. One of the reasons could be that in WILMS a broad range of mRNAsi (Figure 3b) and histologies is observed, from blastemal tumor areas associated with a high mRNAsi and sarcomatous or epithelial differentiated areas associated with lower mRNAsi.

As this is an exploratory study, the results are only correlative in nature. Transcriptome based findings will need to be expanded including scRNAseq analysis with emphasis on specific tumor types. A technical limitation to assess all immune cell types analyzed by transcriptome studies is often the scarcity of tumor tissue. Novel tissue sparing multiplex staining techniques [31] may be helpful in validating CIBERSORT data on a wide range of tumor types and add information as to spatial localization. Furthermore, larger clinical cohorts and longitudinal studies correlating molecular as well as immune TME data will be required to confirm the findings and design clinical scoring systems.

While prognostication of genes overall was similar in adult and pediatric tumor entities, subtle variations were observed when comparing prognostication of abundance of immune cell types in pediatric tumor entities. For instance, in contrast to other tumors in NB M2 type macrophages were associated with longer survival, most likely attributable to similarity in the signature of M2 macrophages and microglia (Figure 2c) and plasma cells correlated with shortened survival. Furthermore, CIBERSORT analysis revealed that CD8+ T-cells in OSTEO predict a poor prognosis (Figure 2c). These results are interesting and will need further functional analysis and clinical validation.

More recently, extensive analysis of pediatric CNS tumors using methyl-CIBERSORT and genomic data did show correlations of the immune TME and molecular subgroups, mutations and prognosis [25], thus the immune TME may depend on specific molecular tumor subtypes. Whether future clinical studies will aim to analyze both, the molecular and immune TME will depend on accompanying trials using immunotherapy and the need to triage the patients prior to multiple treatment options.

What is interesting is a closer look at WILMS. Here, suppression of M2 macrophages are abundant in sarcomatous areas but are devoid in blastemal areas (Figure 4a), a finding that is consistent with earlier immunohistochemistry studies [32]. Because infiltrating M2 type macrophages are strongly connected with the histologic pattern correlating within the same tumor, heterogeneity of the immune stroma appears to be regulated on a microanatomic level and not governed by systemic immune changes. Interestingly, there is possibly a regulatory link in the other direction as well, with M2 macrophages redirecting tumor cells to undergo a stemness program [24,33].

It would be interesting to identify the molecular and structural factors regulating cell entry or exclusion in such tumors in order to render immunotherapeutic resistant tumors accessible.

## 4. Materials and Methods

### 4.1. Gene Expression Data and Study Design

In this study, gene expression microarray data from public archives of 4068 tumors occurring mostly at young age across twelve “pediatric” malignancies were analyzed. Gene expression profiles (GEPs) and clinical information were retrieved from Gene Expression Omnibus (GEO) [34] and corresponding publications selecting only tumors occurring mostly at the pediatric and young adult age. The entire cohort of pediatric tumors was divided into two sub-cohorts (a.) PRECOG-cohort (*n* = 3094), consisting of all tumor samples with available overall survival (OS) data, (b.) CIBERSORT-cohort (*n* = 1883) restricted to the following two microarray platforms Affymetrix HG-U133A (GEO accession number GPL96) and Affymetrix HG-U133 Plus 2.0 (GEO accession number GPL570) to limit batch effects. Patients’ characteristics and microarray datasets used in this study are summarized in Tables S1 and S2 (PRECOG- and CIBERSORT-cohort, respectively). A total of 909 transcriptomes were overlapping in both cohorts. No specific age cutoff was used. Within the CIBERSORT-cohort, the median pediatric age was 10 years (*n* = 1181).

### 4.2. Assessment of Prognostic Landscape by PRECOG

By using PRECOG (PREdiction of Clinical Outcomes from Genomic, http://precog.stanford.edu, accessed on 21 January 2020) we assessed the prognostic landscape of genes across pediatric malignancies compared to adult tumors as described before [27]. We compared our cohort of tumors occurring predominantly at pediatric and young adult age (PRECOG-cohort, Table S1) with a previously published cohort described by Gentles et al. in 2015 consisting of 34 malignancies, including ~18,000 samples with overall survival data from 165 adult tumor type gene expression datasets. Assessment of prognostic associations for all genes following the approach used in PRECOG [27] yielded meta z-scores for each gene per tumor type and a global meta-z-score for all pediatric tumor types combined (Table S3) [27]. The z-score encodes the statistical robustness of the association, but retains the directionality (favorable vs adverse), unlike *p*-values. Data pre-processing and normalization within the PRECOG-cohort was performed as described earlier [27]. Each dataset, regardless of platform, was quantile normalized separately. Associations between prognostic meta-z-scores were assessed by principal component analysis (PCA) (Figure 1a). We did not correct for potential batch effects for PCA analysis and global z-score comparisons. To identify groups of genes with similar prognostic patterns in pediatric versus adult cancers, we plotted the global meta-z-scores of all pediatric tumor entities against the global meta-z-scores of all adult tumors (Figure 1b). Within the dot plot, two sectors were selected, defined by the maximum (adverse genes) and minimum (favorable genes) values of both global meta-z-values (Figure 1b). For each sector, we applied gene ontology (GO) enrichment using hypergeometric testing based on the GO biological pathways database using the R package “clusterprofiler” (version 3.14.3). For enrichment, the genes in each sector were used as an input, significantly enriched terms were defined with a Bonferroni corrected *p*-value cutoff of 0.05 and a FDR cutoff of 0.05. Enriched terms were dot plotted ordered by count size (Figure 1c,d).

### 4.3. Assessment of Immune Infiltration by CIBERSORT

We used CIBERSORT [28] to examine the relative fractions of 22 infiltrating immune cell types in each tumor tissue, using the LM22 signature matrix with 1000 permutations (other parameters were left at default values). The LM22 matrix includes naïve and memory B cells, plasma cells, seven T-cell types (CD8 T-cells, naïve CD4 T-cells, resting memory CD4 T-cells, activated memory CD4 T-cells, follicular helper T-cells, regulatory T-cells, gamma delta T-cells), resting and activated natural killer (NK) cells, monocytes, macrophages (M0 macrophages, M1 macrophages, M2 macrophages), resting and activated dendritic cells (DC), resting and activated mast cells, eosinophils and neutrophils. The sum of all evaluated immune cell type fractions equals one for each tumor sample, hence all estimates are relative to total leukocyte content. GEPs of the CIBERSORT-cohort were restricted to microarray platforms Affymetrix HG-U133A (GEO accession number GPL96) and Affymetrix HG-U133 Plus 2.0 (GEO accession number GPL570), because the leucocyte signature comparison matrix used within the CIBERSORT algorithm was validated on the above platforms [28]. Affymetrix array data was downloaded as CEL files (raw data) from GEO and probes were aggregated to HUGO gene symbols. All microarray studies were normalized according to the “Robust Multi-array Average” (RMA) method [35] prior to analysis using the “affy” package in Bioconductor and R (R Foundation for Statistical Computing). CIBERSORT produces an empirical *p*-value for the deconvolution using Monte Carlo sampling. GEPs with a CIBERSORT *p*-value less than 0.05 were used for further analysis. 

### 4.4. Calculation of Gene Expression-Based Stemness Index (mRNAsi)

For all samples within the CIBERSORT-cohort, we calculated the gene expression-based stemness index mRNAsi [4]. This stemness index is a predictive model using an OCLR algorithm on pluripotent stem cell samples from the Progenitor Cell Biology Consortium dataset [36,37] to train the stemness signature. The mRNA expression-based signature contains a gene expression profile comprising 11,774 genes. The workflow to generate the mRNAsi is available on https://bioinformaticsfmrp.github.io/PanCanStem_Web/ (accessed on 30 September 2019). We applied the stemness index model to score all samples within the CIBERSORT-cohort. RMA- and log2 transformed normalized values of gene expression data were used to generate the mRNAsi. The stemness index was subsequently mapped to the (0,1) range.

### 4.5. Tissue Microarray (TMA) Cohort, Immunohistochemistry and Scoring

A tumor tissue microarray (TMA) consisting of 78 WILMS tumor tissue specimens was generated according to the local ethics board regulation (University of Homburg-Saar, collaboration Norbert Graf) and the international declaration of Helsinki. TMA construction was performed as described earlier [38]. Briefly, formalin-fixed, paraffin-embedded tissue samples were used for constructing TMAs. Two 0.5 mm cores from the tumor-containing donor blocks were inserted into a recipient paraffin block. To represent carcinomatous tissue sufficiently, fixed paraffin blocks containing different areas of the tumor (blastemal, epithelial and stromal areas) were used for sampling.

For immunohistochemistry, TMA and standard paraffin sections (2–3 μm) were dried at 65 °C and placed in 200 mL of target retrieval solution (citrate buffer pH 6.0, Medac PMB 1–250). Afterwards, sections were washed with Tris-buffered saline (Medac B1–30A), then with distilled water. Endogenous peroxidase was blocked using H2O2. IHC was performed with primary antibodies against CD163 (clone MRQ-26, Medac, 1:1000) and developed using a DAB IHC Detection system on a semi-automatic immunohistochemistry stainer (Autostainer 480S; Medac, Wedel, Germany). Slides were reviewed and scored by two board-certified pathologists (IG, CV). Per core, the main histologic pattern (blastemal, epithelial, stromal) was assessed and an average score of 1–2 high power fields (HPFs) counted. Regressed tumor stroma was excluded from quantitative analysis. In total, tissue specimens of 78 patients within 134 TMA cores could be analyzed. Photomicrographs were taken with a BX51 microscope (Olympus, Hamburg, Germany) and a Zeiss AxioCam MRc5 camera using the Axiovision software (Carl Zeiss, Oberkochen, Germany).

### 4.6. Statistical Analysis

Statistical analysis was performed using the software package IBM SPSS (Chicago, IL, USA) Statistics for Windows (version 24) and R (version 4.0.0). Mean value comparisons were performed with the Mann–Whitney U and Kruskal–Wallis test depending on the number of compared groups. The Benjamini–Hochberg (BH) procedure was used to correct for multiple testing errors with a significance rate of 0.05, where indicated [39]. If not otherwise specified in the figure legends, data are presented as box plots with horizontal bars representing the median. *p*-values for the associations between stemness indices, the pediatric immune microenvironment and gene expression were computed using Spearman’s correlation coefficient tests followed by multiple testing using the BH method. Survival analyses were performed using the Kaplan–Meier method and the log-rank test. Cutoff points of T-cell abundance expressed as the percentage of cell fraction within all immune cells were set at the point with the most significant (log-rank test) separation (minimum *p*-value method) using the web-based tool “cutoff finder” [29]. Cutoffs for mRNAsi Kaplan–Meier analysis were based on quartiles as indicated. Univariate and multivariate analysis were performed using Cox regression analysis using the R package “survival” implemented coxph function (version 3.1.12). Hazard ratios (HR) and their 95% confidence intervals (95% CI) were calculated. *p*-values less than 0.05 were considered statistically significant.

## 5. Conclusions

This study gives detailed insight into the prognostic landscape of gene expression, infiltrating immune cells and stemness using 4068 transcriptomes of tumors occurring predominantly at the pediatric and young adult age. It can be used as a resource for the identification and characterization of molecular and cellular biomarkers. We demonstrate a correlation of stemness with precursor cell morphology, molecular and clinical subtype, but not with a distinct pattern of the immune TME defined by CIBERSORT. However, stemness correlates with selected immune cell types in WILMS on a microanatomic level indicating heterogeneity of the immune TME and locoregional, not systemic regulation. Prospectively, it would be interesting to define the molecular and cellular mechanisms regulating this microanatomic crosstalk in order to improve immunotherapy in pediatric tumors.

## Data Availability

Only publicly available datasets were analyzed in this study. Accession numbers can be found in Tables S1 and S2.

## Supplementary Materials

The following are available online at https://www.mdpi.com/2072-6694/13/4/854/s1; Table S1. PRECOG-cohort. 12 pediatric tumor types; Table S2. CIBERSORT-cohort. 11 pediatric tumor types. The CIBERSORT cohort was restricted to Affymetrix platforms (HGU133Plus2 [GPL570] and HGU133A [GPL96]), as the leucocyte signature matrix used within the CIBERSORT algorithm was validated on the above platforms [28]. Indicated clinical data was retrieved from Gene Expression Omnibus (GEO) and published literature; Table S3. PRECOG z-scores per single gene and pediatric tumor entity; Table S4. Top 10 adverse and favorable prognostic genes. Pediatric PRECOG-cohort. Genes with highest and lowest prognostic meta-z-scores (OS) per tumor entity; Table S5. Univariate and multivariate Cox regression analysis in NB; Table S6. Correlation of mRNAsi with relative proportions of immune cell subsets (CIBERSORT) in Wilms tumor. Naïve B-cells, CD8+ T-cells, follicular helper T-cells, regulatory T-cells, monocytes and activated mast cells correlated directly (green) and CD4 memory T-cells, gamma delta T-cells, M0, M1 and M2 type macrophages, resting mast cells and eosinophils correlated indirectly (red) with the mRNAsi. Significant differences after correcting for multiple testing error using the Benjamini–Hochberg (BH) procedure are indicated in bold. Spearman rank test.
