# Supplementary material for: Prognostic Gene Expression, Stemness and Immune Microenvironment in Pediatric Tumors
Source: Cancers (Basel). 2021 Feb 18;13(4):854. doi: 10.3390/cancers13040854 (PMC7922568; doi:10.3390/cancers13040854)
Supplement: Supplementary file 1 [file cancers-13-00854-s001.zip › supplementary/cancers-1113386-supplementary.docx]

Supplementary Materials

Prognostic Gene Expression, Stemness and Immune Microenvironment in Pediatric Tumors

**Supplementary Table 1.** PRECOG-cohort. 12 pediatric tumor types.

Abbreviations: acute lymphatic leukemia, ALL; atypical teratoid rhabdoid tumor, ATRT; Ewing’s sarcoma family of tumors, ESFT; germ cell tumors, GCT; high grade gliomas, HGG; medulloblastomas, MB; neuroblastoma, NB; osteosarcoma, OSTEO; CNS-PNETs; rhabdomyosarcoma, RMS; synovial sarcomas, SS; Wilms tumors, WILMS. Overall survival, OS.

| **data set** | **platform** | **cancer type** | **outcome** | **no. samples** |
| --- | --- | --- | --- | --- |
| GSE11877 | HGU133Plus2 | ALL | OS | 207 |
| GSE14286 | HGU133A | ALL | OS | 13 |
| GSE28026 | HGU133Plus2 | ATRT | OS | 17 |
| GSE34620 | HGU133Plus2 | ESFT | OS | 38 |
| GSE63157 | GPL5175 | ESFT | OS | 85 |
| GSE17679 | HGU133Plus2 | ESFT | OS | 32 |
| GSE8303 | GPL2891 | ESFT | OS | 12 |
| GSE10783 | HGU133AB | GCT | OS | 34 |
| GSE3218 | HGU133AB | GCT | OS | 74 |
| GSE19348 | HGU133Plus2 | GCT | OS | 13 |
| GSE19578 | HGU133Plus2 | HGG | OS | 47 |
| Pomeroy_CNS | GPL80 | MB | OS | 60 |
| GSE30074 | GPL6244 | MB | OS | 30 |
| GSE50765 | GPL11532 | MB | OS | 68 |
| GSE85217 | GPL22286 | MB | OS | 612 |
| Berwanger_NB | Berwanger | NB | OS | 85 |
| E-MTAB-179 | NBCustomAgilent4x44K | NB | OS | 478 |
| E-TABM-38 | A-MEXP-255 | NB | OS | 251 |
| GSE27608 | GPL5188 | NB | OS | 47 |
| GSE12460 | HGU133Plus2 | NB | OS | 52 |
| GSE13136 | HGU133Plus2 | NB | OS | 29 |
| GSE16476 | HGU133Plus2 | NB | OS | 88 |
| GSE16091 | HGU133A | OSTEO | OS | 34 |
| GSE21257 | GPL10295 | OSTEO | OS | 53 |
| GSE39055 | GPL14951 | OSTEO | OS | 37 |
| GSE14295 | GPL6102 | CNS-PNET | OS | 19 |
| E-TABM-1202 | HGU133Plus2 | RMA | OS | 101 |
| ca0099 | HGU133A | RMA | OS | 145 |
| GSE20196 | HGU133Plus2 | SS | OS | 34 |
| GSE10320 | HGU133A | WILMS | OS | 65 |
| GSE14767 | HGU133A | WILMS | OS | 10 |
| GSE31403 | HGU133A | WILMS | OS | 224 |
| **TOTAL** |  | 12 malignancies |  | **3,094** |

**Supplementary Table 2.** CIBERSORT-cohort. 11 pediatric tumor types. The CIBERSORT cohort was restricted to Affymetrix platforms (HGU133Plus2 [GPL570] and HGU133A [GPL96]), as the leucocyte signature matrix used within the CIBERSORT algorithm was validated on the above platforms [28]. Indicated clinical data was retrieved from Gene Expression Omnibus (GEO) and published literature.

Abbreviations: acute lymphatic leukemia, ALL; atypical teratoid rhabdoid tumor, ATRT; Ewing’s sarcoma family of tumors, ESFT; germ cell tumors, GCT; high grade gliomas, HGG; medulloblastomas, MB; neuroblastoma, NB; osteosarcoma, OSTEO; CNS-PNETs; rhabdomyosarcoma, RMS; synovial sarcomas, SS; Wilms tumors, WILMS. Overall survival, OS; event-free survival, EFS; relapse-free survival, RFS.

| **data set** | **Platform** | **cancer type** | **clinical data** | **no. samples** |
| --- | --- | --- | --- | --- |
| GSE19404 | GPL570 | ATRT | NA | 1 |
| GSE35493 | GPL570 | ATRT | NA | 2 |
| GSE65132 | GPL570 | ATRT | gender | 7 |
| GSE28026 | GPL570 | ATRT | age, gender, OS | 18 |
| GSE70678 | GPL570 | ATRT | age, gender, subgroup | 49 |
| GSE19404 | GPL570 | CNS-PNET | NA | 13 |
| GSE74195 | GPL570 | CNS-PNET | NA | 5 |
| GSE35493 | GPL570 | CNS-PNET | NA | 9 |
| GSE73038 | GPL570 | CNS-PNET | age, gender, OS, PFS | 59 |
| GSE68015 | GPL570 | CNS-PNET | NA | 4 |
| GSE1825 | GPL96 | ESFT | NA | 5 |
| GSE37371 | GPL96/570 | ESFT | NA | 39 |
| GSE34620 | GPL570 | ESFT | OS, EFS, age | 117 |
| GSE17679 | GPL570 | ESFT | OS, EFS, age, gender | 32 |
| GSE15757 | GPL96 | ESFT | NA | 4 |
| GSE10783 | GPL96 | GCT | subgroup | 34 |
| GSE18155 | GPL96 | GCT | subgroup | 3 |
| GSE10615 | GPL96 | GCT | subgroup | 27 |
| GSE19348 | GPL570 | GCT | CNS_GCT | 13 |
| GSE3218 | GPL96 | GCT | subgroup | 91 |
| GSE35493 | GPL570 | HGG | NA | 12 |
| GSE73038 | GPL570 | HGG | age, gender | 18 |
| GSE36245 | GPL570 | HGG | age, gender, PFS, OS, subgroup | 46 |
| GSE19578 | GPL570 | HGG | age, OS | 33 |
| GSE66354 | GPL570 | HGG | NA | 14 |
| GSE66354 | GPL570 | MB | subgroup | 3 |
| GSE19404 | GPL570 | MB | NA | 8 |
| GSE37418 | GPL570 | MB | age, gender, subgroup, histology | 76 |
| GSE74195 | GPL570 | MB | NA | 25 |
| GSE67850 | GPL570 | MB | age, gender, histology | 22 |
| GSE35493 | GPL570 | MB | subgroup | 21 |
| GSE12992 | GPL570 | MB | age, histology | 40 |
| GSE10327 | GPL570 | MB | age, gender, histology | 62 |
| GSE73038 | GPL570 | MB | age, gender, OS, PFS, subgroup | 47 |
| GSE68015 | GPL570 | MB | NA | 1 |
| GSE1825 | GPL96 | NB | NA | 5 |
| GSE16476 | GPL570 | NB | age, gender, PFS, OS, MYC, INSS | 88 |
| GSE16237 | GPL570 | NB | age, MYC, INSS | 50 |
| GSE12460 | GPL570 | NB | age, gender, PFS, OS, MYC, INSS | 53 |
| GSE13136 | GPL570 | NB | age, gender, OS, MYC, INSS | 30 |
| GSE14359 | GPL96 | OSTEO | age, gender, grade | 5 |
| GSE14827 | GPL570 | OSTEO | age, gender, histology | 27 |
| GSE16088 | GPL96 | OSTEO | NA | 14 |
| GSE16091 | GPL96 | OSTEO | OS | 34 |
| GSE66533 | GPL570 | RMS | fusion status | 58 |
| GSE92689 | GPL96 | RMS | age, OS, histology | 158 |
| GSE68015 | GPL570 | RMS | NA | 8 |
| GSE20196 | GPL570 | SS | age, gender, OS, histology | 34 |
| GSE53224 | GPL570 | WILMS | histology, gender | 53 |
| GSE31403 | GPL96 | WILMS | histology, stage, age, OS, RFS | 224 |
| GSE11482 | GPL96 | WILMS | histology, stage, age | 3 |
| GSE14767 | GPL96 | WILMS | histology, stage, age, OS, RFS | 11 |
| GSE10320 | GPL96 | WILMS | histology, stage, age, OS, RFS | 68 |
| **TOTAL** |  | 11 malignancies |  | **1,883** |
| OS, overall survival; RFS, relapse-free survival; PFS, progression-free survival; NA, not applicable. | | | | |

**Supplementary Table 3.** PRECOG z-scores per single gene and pediatric tumor entity.

Please find attached.

**Supplementary Table 4.** Top 10 adverse and favorable prognostic genes. Pediatric PRECOG-cohort. Genes with highest and lowest prognostic meta-z-scores (OS) per tumor entity.

Please find attached.

**Supplementary Table 5.** Univariate and multivariate Cox regression analysis in NB.

| N=162 |  | **Overall survival** | |  |
| --- | --- | --- | --- | --- |
| Parameter | Category | HR | 95% CI | *p* |
| Univariate |  |  |  |  |
| INSS | I/II/III/IVS vs. IV | 2.339 | 1.794-3.051 | **<0.001** |
| MYCN-status | amplified vs. non-amplified | 0.180 | 0.104-0.310 | **<0.001** |
| mRNAsi | low vs. high (median cut-off) | 4.834 | 2.544-9.186 | **<0.001** |
| Multivariate |  |  |  |  |
| INSS | I/II/III/IVS vs. IV | 1.896 | 1.419-2.535 | **<0.001** |
| MYCN-status | amplified vs. non-amplified | 0.405 | 0.230-0.710 | **0.002** |
| mRNAsi | low vs. high (median cut-off) | 2.086 | 1.033-4.213 | **0.04** |

**Supplementary Table 6.** Correlation of mRNAsi with relative proportions of immune cell subsets (CIBERSORT) in Wilms tumor. Naïve B-cells, CD8+ T-cells, follicular helper T-cells, regulatory T-cells, monocytes and activated mast cells correlated directly (green) and CD4 memory T-cells, gamma delta T-cells, M0, M1 and M2 type macrophages, resting mast cells and eosinophils correlated indirectly (red) with the mRNAsi. Significant differences after correcting for multiple testing error using the Benjamini-Hochberg (BH) procedure are indicated in bold. Spearman rank test.

| **Immune cell subset (CIBERSORT)** | **Spearman´s rho** | **BH corrected *p*-value** |
| --- | --- | --- |
| naive B-cells | 0.26 | **0.0009** |
| memory B-cells | -0.05 | 0.5657 |
| plasma cells | -0.02 | 0.8067 |
| CD8+ T-cells | 0.34 | **1.62 x 10^-5^** |
| CD4+ naive T-cells | NA | NA |
| CD4+ memory resting T-cells | -0.31 | **0.0001** |
| CD4+ memory activated T-cells | -0.32 | **6.74 x 10^-5^** |
| follicular helper T-cells | 0.25 | **0.0017** |
| regulatory T-cells | 0.44 | **8.84 x 10^-9^** |
| gamma delta T-cells | -0.42 | **6.6 x 10^-8^** |
| resting NK cells | 0.09 | 0.3217 |
| activated NK cells | 0.04 | 0.6824 |
| monocytes | 0.31 | **8.83 x 10^-5^** |
| M0 macrophages | -0.28 | **0.0004** |
| M1 macrophages | -0.38 | **1.04 x 10^-6^** |
| M2 macrophages | -0.51 | **1.81 x 10^-11^** |
| resting dendritic cells | -0.02 | 0.8067 |
| activated dendritic cells | 0.16 | 0.0455 |
| resting mast cells | -0.44 | **8.84 x 10^-9^** |
| activated mast cells | 0.54 | **5.19 x 10^-13^** |
| eosinophils | -0.46 | **2.59 x 10^-9^** |
| neutrophils | -0.06 | 0.5656 |
